# Mammalian mitochondrial iron–sulfur cluster biogenesis and transfer and related human diseases

**DOI:** 10.52601/bpr.2021.200038

**Published:** 2021-04-30

**Authors:** Wenxin Zhang, Li Xu, Hongting Zhao, Kuanyu Li

**Affiliations:** 1 Medical School of Nanjing University, Jiangsu Key Laboratory of Molecular Medicine, Nanjing 210093, China

**Keywords:** Mitochondria, Fe–S cluster synthesis and transfer, Congenital sideroblastic anemia, Neurodegenerative diseases

## Abstract

As a cofactor, iron–sulfur (Fe–S) cluster binds to proteins or enzymes that play important roles in various important biological processes, including DNA synthesis and repair, mitochondrial function, gene transcription and translation. In mammals, the core components involved in Fe–S cluster biosynthesis are considered to include the scaffold protein ISCU, cysteine desulfurase NFS1 and its accessory proteins ISD11 and ACP, and frataxin (FXN). Proteins involved in Fe–S cluster transfer have been found to include HSC20/HSPA9, as chaperone system, and Fe–S cluster carriers. The biosynthesis and transfer of Fe–S clusters to Fe–S recipients require fine-tune regulation. Recently, significant progress has been made in the structure and mechanism of mitochondrial Fe–S biosynthesis and transfer. Based on, especially, the development of DNA sequencing technology, bioinformatics, and gene editing technology, diseases caused by mutations of Fe–S cluster-related genes have been revealed in recent years, promoting the rapid development in the field of Fe–S and human health. This review focuses on the function of genes involved in Fe–S cluster biosynthesis and transfer and on the diseases caused by the mutations of the related genes. Finally, some questions we are facing are raised, new hypotheses presented, and the perspectives discussed.

## INTRODUCTION

**Figure 1:**
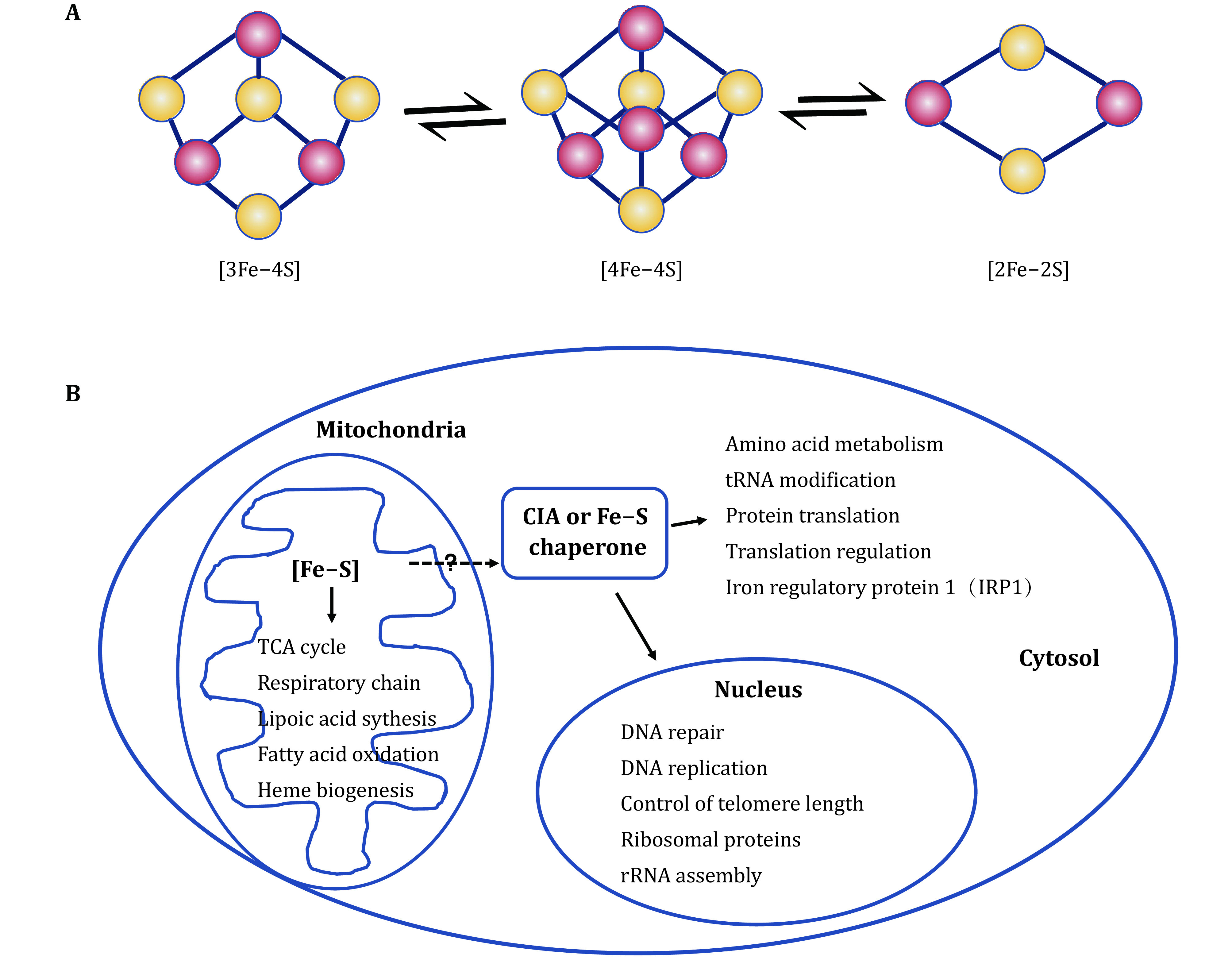
The repertoire of Fe–S proteins impacting numerous cellular processes. **A** The basic Fe–S clusters and their interconversion. **B** Fe–S proteins involved in an astonishing array of reactions. The extra-mitochondrial Fe–S machinery (cytosolic iron–sulfur assembly, CIA) might, to some extent, rely on the mitochondrial system

Iron–sulfur cluster (Fe–S) is a cofactor composed of inorganic iron and sulfur (Fig. 1A), which almost does not exist alone. It often binds to cysteine residues of proteins, occasionally histidine, serine or aspartic acid, to help gaining enzymatic activity or acting as a redox sensor. Fe–S cluster is thought to be one of the oldest form of cofactors and existed before life. In recent years, Fe–S cluster containing proteins have been found to be involved in various metabolic pathways and physiological activities in mammals, including many classical biochemical pathways such as respiratory chain, citric acid cycle, heme biogenesis, oxidation of fatty acids, synthesis of lipoic acid and biotin in mitochondria, and the recently discovered roles in RNA and DNA metabolism (Fig. 1B) (Braymer and Lill 2017; Fuss *et al*. 2015; Maio *et al*. 2020). The instability of Fe–S clusters *in vitro* includes the sensitivity to oxygen, so early studies did not pay enough attention to the existence of Fe–S clusters along with proteins. With the emergence of new methods and the continuous development of technology, especially in conjunction with the development of bioinformatics, more and more Fe–S proteins have been identified. Fe–S clusters in biochemical reactions often reflect the redox state of the cells by accepting or donating a single electron, stabilizing the Fe–S proteins, or directly regulating the redox reaction basing on the active chemical properties of iron. Besides, ferrochelatase (FECH), a terminal enzyme in heme biosynthesis, catalyzes the insertion of ferrous iron into protoporphyrin IX to form heme in mammals when FECH obtains 2Fe–2S. Therefore, the function and importance of Fe–S proteins have received more and more attention.

**Figure 2:**
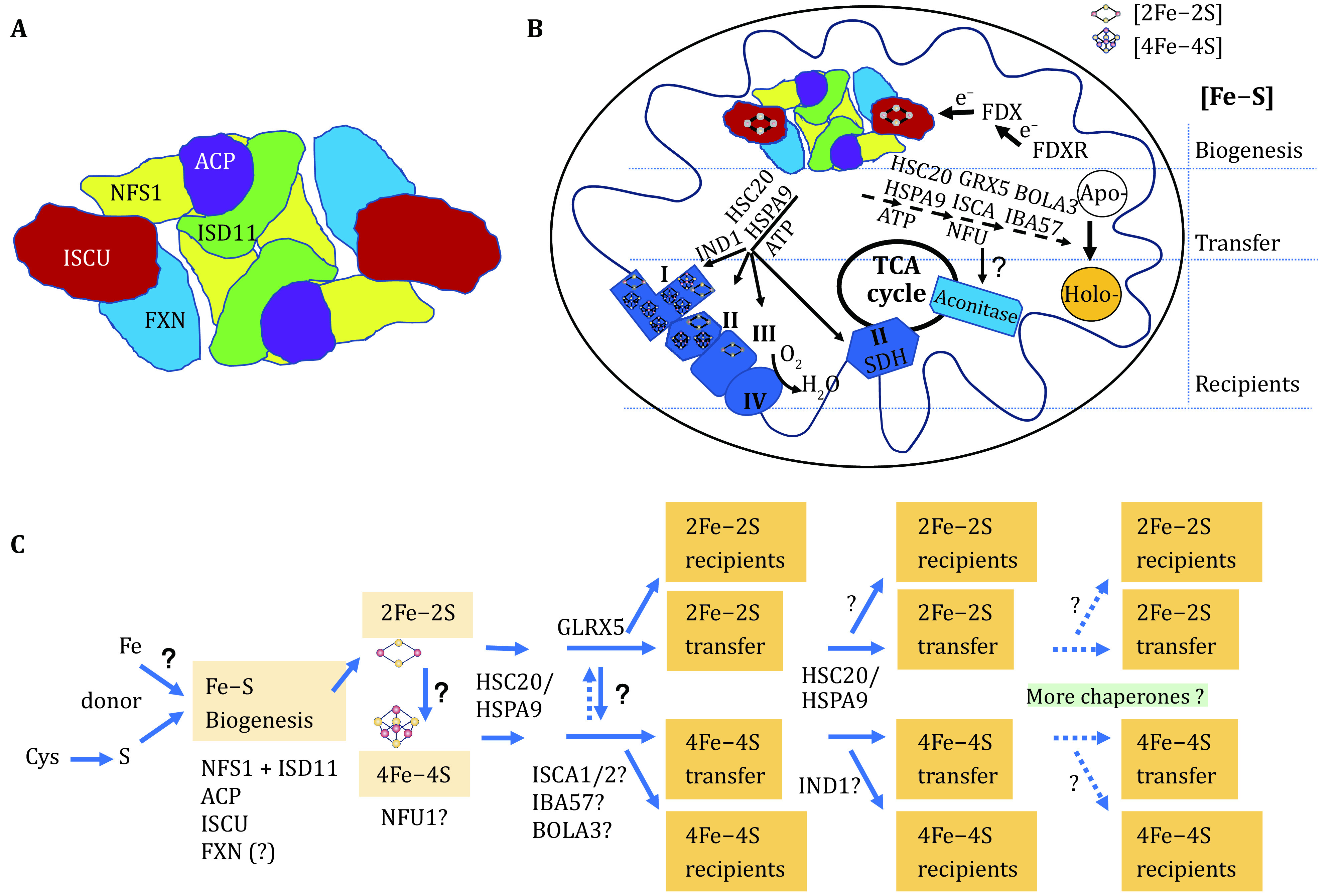
A model of the highly conserved Fe–S biogenesis pathway and putative transfer system in mammalian mitochondria. **A** A simplified structure of the core components of Fe–S biogenesis machinery showing (NFS1 + ISD11):ACP:ISCU:FXN=2:2:2:2. Adapted from Fox *et al*. (2019) and Cai *et al*. (2018a). **B** Mitochondrial biogenesis and transfer of Fe–S clusters. **C** A proposed hierarchy structure of mitochondrial Fe–S cluster biosynthesis and transfer

The biosynthetic pathways of Fe–S clusters are quite conservative in view of an evolutionary perspective. From prokaryotes to mammals, the core proteins, cysteine desulfurase (in human, abbreviation NFS1) and scaffold protein (ISCU), are sequence-and-function conserved. A non-essential CyaY protein in low organisms (such as *Escherichia coli*) is a homolog of mammalian frataxin (FXN), which is essentially required for embryogenesis (Cossee *et al*. 2000). ISD11 and ACP (an acyl carrier protein), two accessory proteins, increase the stability of NFS1 and the efficiency of Fe–S cluster biosynthesis. ISD11, also known as LYRM4, harbors LYR (leucine-tyrosine-arginine) motif and forms a stable complex with NFS1 and ACP (Adam *et al*. 2006; Cory *et al*. 2017). It is currently believed that NFS1, ISD11, ACP, ISCU and FXN constitute the mitochondrial core components of mammalian Fe–S cluster machinery. By expressing these components *in vitro*, the structural biology has demonstrated that the ratio of NFS1 + ISD11, ACP, ISCU and FXN is 2:2:2:2 (Fox *et al*. 2019) (Fig. 2A).

To date, scientists believe that most Fe–S clusters are synthesized in mitochondria. It is controversial whether the extra-mitochondrial Fe–S clusters are independently synthesized outside mitochondria or indirectly derived from mitochondria. It seems that the extra-mitochondrial Fe–S proteins are, to some extent, dependent on mitochondrial machinery. Therefore, the extra-mitochondrial (mostly, called cytosolic) iron–sulfur assembly machinery (CIA) is supposed to mainly stabilize and repair Fe–S proteins/enzymes for functionality.

Insufficient synthesis of Fe–S clusters or uncontrollable disintegration is detrimental to cells. The mutations of genes *NFS1, ISD11, FXN* or *ISCU* result in insufficient synthesis of Fe–S clusters, which causes a number of diseases with cellular deficit of ATP. However, the mutation of genes participating the transfer of Fe–S clusters presents distinct clinical manifestation, implying that the transfer of Fe–S clusters is in a hierarchical structure, in which Fe–S carrier proteins target their own recipients (Fig. 2, see the detailed description later).

## MITOCHONDRIAL Fe–S BIOGENESIS IN MAMMALS

Fe–S protein has attracted the attention of scientists only in the past 30 years. The research on the synthesis of Fe–S clusters started with nitrogenase in nitrogen-fixing bacteria. With the development of new methods/technologies and understanding of biological genomes, it has been expanding to other prokaryotes and eukaryotes rapidly.

In eukaryotes, the earliest known Fe–S proteins were mitochondrial aconitase and succinate dehydrogenase. The great progress has been made over the past 20 years on mammalian Fe–S cluster biogenesis. Because of the conservative feature of Fe–S cluster synthesis evolutionarily, most of the genes involved in Fe–S cluster synthesis and transfer were revealed by homology searching from prokaryotes to eukaryotic yeast and mammals. The first identified were the most important proteins NFS1 and ISCU in mammals (Land and Rouault 1998; Tong and Rouault 2000). Although the full details of the synthesis of Fe–S clusters have not yet been completely clarified, the basic framework has been recognized, and there are more than 30 proteins known to participate in the synthesis and delivery of Fe–S clusters (Braymer and Lill 2017). It is interesting that low organisms (prokaryotes) have three distinct systems including iron–sulfur cluster (ISC), sulfur mobilization (SUF), and nitrogen fixation (NIF), which are used under basic conditions, oxidative stress conditions and nitrogen-fixing conditions, respectively, for Fe–S cluster synthesis. *E. coli* possesses at least the first two systems. However, mammals have only kept one constitutive ISC system. Therefore, mutations in genes that affect the synthesis of Fe–S clusters would increase the risk of diseases when there is no alternative pathway or function of compensation genes in human.

Fe–S clusters can self-assemble with low efficiency under *in vitro* conditions without oxygen and with iron and sulfur ions, whereas the catalytic effect of the cysteine desulfurase (NFS1 + ISD11 complex) greatly improves the efficiency of Fe–S cluster synthesis on the scaffold ISCU (Land and Rouault 1998; Li *et al*. 2006). The effect of necessary protein FXN *in vitro* is not as important as it *in vivo*, probably, due to the high concentration of iron ion and cysteine *in vitro* (not published). The biosynthesis of Fe–S clusters in mitochondria is strictly enzyme-catalyzed and initiates with the activity of NFS1, which is dependent on pyridoxal 5'-phosphate (PLP) as a cofactor. Cysteine is desulfurized to generate alanine, while the removed sulfur (S0) receives electrons and is ligated with cysteine of NFS1 through a disulfide bond to produce a persulfide NFS1 intermediate (NFS1- Cys381-SH) (Patra and Barondeau 2019). ISD11 plays a role in assisting and stabilizing NFS1, which contributes to the formation of persulfide NFS1 (Adam *et al*. 2006; Shi *et al*. 2009) and the transfer of sulfur to iron-holding ISCU (Lin *et al*. 2020). The function of ACP is not very clear, but it can interact with ISD11, independent on NFS1 *in vitro* and for a stable assembly complex *in vivo* (Cory *et al*. 2017). Though how ISCU gets iron is unknown, 2Fe-2S is considered to be assembled first transiently on ISCU depending on the function of NFS1/ISD11/ACP complex (Maio *et al*. 2020). FXN can interact with ISCU and NFS1 (Bridwell-Rabb *et al*. 2014) to facilitate the enzymatic activity of NFS1 as an allosteric activator (Patra and Barondeau 2019). Interestingly, just one amino acid substitution of IscU (M141I) in yeast can make the strain survive in an FXN-independent manner (Yoon *et al*. 2015). Based on the interaction between FXN and ISCU, it is speculated that the interaction between FXN and ISCU changes the conformation of ISCU, resulting in the easy access of -S onto ISCU from NFS1 for Fe–S cluster formation, which is partially supported by previous report (Cai *et al*. 2018b). FXN binds at the interface of two NFS1 and one ISCU subunits, modifying the local environment of a bound zinc ion that would otherwise inhibit NFS1 activity in complexes without FXN (Fox *et al*. 2019). As a result, FXN binding accelerates or pushes the whole assembly process. The synthesized 2Fe–2S is then converted by a certain allosteric or catalytic protein into 4Fe–4S, likely, on a 4Fe–4S scaffold, for instance NFU1, under the condition of consuming ATP. It seems that the loss of FXN does not affect the activity of the 2Fe–2S protein, *e.g*. FECH, but severely the activities of 4Fe–4S, *e.g*. aconitase. Then, we ask whether FXN plays an important role in the convert from 2Fe–2S to 4Fe–4S (Li 2019). *In vitro* experiments supported this speculation (Colin *et al*. 2013). The recipients can directly or indirectly obtain 2Fe–2S or 4Fe–4S from scaffold or carriers with the assistance of the chaperone proteins HSPA9/HSC20 and Fe–S transfer protein, which makes the recipients gain the function (Fig. 2).

The discovery of two accessory components, ISD11 and ACP, of the synthetic complex in mammals is not due to the study of prokaryotes, but of yeast, of which ISD11 has no homologue in prokaryotes (Wiedemann *et al*. 2006) and ACP has (Van Vranken *et al*. 2016). ISD11 interacting with NFS1 significantly affects the stability of NFS1 and the synthesis of Fe–S clusters (Wiedemann *et al*. 2006). Besides, the interaction between ACP and ISD11 does not depend on NFS1, whereas ACP in prokaryotes directly interaction with IscS, homologue of mammalian NFS1. The loss of ACP in eukaryotes prevents the assembly of the entire Fe–S cluster machinery so that *ACP* is an essential gene (Van Vranken *et al*. 2016). ACP is also called NDUFAB1 (NADH: ubiquinone oxidoreductase subunit AB1) as a subunit of mitochondrial complex I. Because it carries acyl group, it also plays an important role in fatty acid metabolism (Feng *et al*. 2009). The mutation of ACP might associate to depression and manic mental illness (Wellcome Trust Case Control 2007), indicating the diversity and importance of the physiological function of ACP.

The biosynthesis of Fe–S clusters requires a stable supply of electrons (e^−^), which is supplied by a special electron supply system, ferredoxin/ferredoxin reductase and NADPH (FDX/FDXR/NADPH), in the early steps of mitochondrial Fe–S cluster biosynthesis (Fig. 2B). During the NFS1 catalytic reaction, the sulfur (S0) from cysteine obtains the electrons, provided by FDX, to be reduced into the sulfide (S^2−^). The human genome contains two homologous FDX1 and FDX2, both of which are important in Fe–S cluster biosynthesis (Shi *et al*. 2012). FDX is an Fe–S protein and contains stable 2Fe–2S, which can even be easily detected after overexpression and purification from *E. coli*. Therefore, efficient synthesis of Fe–S clusters promotes the activity of FDX. Conversely, the synthesis of Fe–S clusters diminishes that results in the reduced activity of FDX and further mitochondrial iron overload and cytoplasmic iron depletion, a common feature that is often observed when Fe–S cluster assembly proteins are deleted (Lill *et al*. 2005; Rouault and Tong 2008).

The extra-mitochondrial synthesis of Fe–S clusters was recognized later than mitochondrial system. Two contrasting models have emerged to describe cytoplasmic Fe–S biogenesis. The first one is a *de novo* cytosolic pathway, independent on mitochondrial machinery (Li *et al*. 2006; Tong and Rouault 2006, 2007). The second one completely relies on mitochondria. The connection between mitochondrial and cytosolic Fe–S cluster is considered to be through a mitochondrial inner-membrane protein ABCB7, which transports an unknown -S containing compound (X–S) (Stehling and Lill 2013). This sulfur-containing compound is probably a 2Fe–2S-containing tetramer of glutathione (Li and Cowan 2015). Once receiving the X–S or 2Fe–2S transported out of the mitochondria, a series of cytoplasmic proteins that participate in or maintain Fe–S clusters begin to assemble and transfer Fe–S to the recipients that require Fe–S clusters (Lill 2020). Both machineries might operate in parallel and the former one is supposed to mainly stabilize and repair Fe–S protein/enzymes for functionality, while the later one contributes to the steady supply of Fe–S cluster.

## REGULATION OF Fe–S BIOGENESIS

Prokaryotes use ISC, SUF or NIF systems for Fe–S biosynthesis to cope with different environmental conditions and meet the needs of Fe–S clusters; but higher organisms, including humans, have only one Fe–S assembly system as we know to date. Therefore, mammalian Fe–S cluster synthesis and post-synthesis stability and transfer require elaborate regulatory mechanisms. Both oxidants (including oxygen) and the oxidizing environment may destroy the Fe–S clusters, consequently, to destabilize the protein or simply make the protein lose the enzymatic activities. Therefore, the cells would adopt various regulatory strategies to adjust the expression of genes involved in biosynthesis of the Fe–S clusters to face the challenge of the environments.

The expression of FXN and ISCU shares a similar response to cellular iron level. Both FXN and ISCU were down-regulated (Li *et al*. 2018) when the availability of functional iron was restricted after iron regulatory protein 2 (IRP2) was knocked out (Jeong *et al*. 2011). Their expression simultaneously increases under high iron conditions (Li *et al*. 2008, 2019; Tong and Rouault 2006). The response to oxygen seems also synchronous but with an unknown mechanism. The expression of hypoxia-inducible factors HIFs (HIF1a and HIF2a) was up-regulated in *IRP2* knockout cells along with the reduced expression of FXN and ISCU, whereas their expression is simultaneously up-regulated when HIFs were suppressed (Li *et al*. 2019). It has been demonstrated that hypoxia reduces ISCU expression through microRNA (miR210), which belongs to the HIF regulon. miR210 can effectively inhibit ISCU translation by binding to the 3'-UTR of *ISCU* mRNA (Chan *et al*. 2009). Except for *FXN* and *ISCU*, there are few studies on the regulation of other Fe–S cluster synthesis-related genes.

In addition to the regulation of gene expression by iron and oxygen, it is clear that the environmental conditions also take important roles in Fe–S clusters themselves. This work is mostly conducted in prokaryotes and few studies in eukaryotes, especially in mammals (Crack and Le Brun 2018). Very likely, there is no significant difference between prokaryotes and eukaryotes. The conversion between 4Fe–4S and 2Fe–2S is shown here (Crack and Le Brun 2018) in presence of oxygen and iron:

Step 1: [4Fe–4S]^2+^ + O_2_ → [3Fe–4S]^1+^ + Fe^2+^ + O_2_^−^

Step 2-1: [3Fe–4S]^1+^ → [2Fe–2S]^2+^ + Fe^3+^ + 2S^2−^

Step 2-2: one persulfide ligand

　[3Fe–4S]^1+^ +O_2_ + 2H^+^ → [2Fe–2S]^2+^ (S) + Fe^3+^ 　　 + S^2−^ + H_2_O_2_

Step 2-3: two persulfide ligands

　[3Fe–4S]^1+^ + O_2_ + 4H^+^ → [2Fe–2S]^2+^ (S)_2_ 　　 + Fe^3+^ + 2H_2_O

Aconitase is an enzyme that contains prosthetic groups, 4Fe–4S, in both prokaryotic and eukaryotic organisms, catalyzing the conversion of citric acid to isocitrate. Only three irons in 4Fe–4S are complexed with cysteines of the protein, and the other iron is the site for binding to the reaction substrate. Therefore, this vacant iron is easily lost in case of oxidative stress, resulting in the conversion of 4Fe–4S to the inactive 3Fe–4S form. The 3Fe–4S form seems to be easily repaired under appropriate conditions depending on the harshness of the circumstance. When the level of oxidative stress is high, 3Fe–4S is further disintegrated to lose the entire Fe–S cluster, which causes the instability of aconitase. The iron regulatory protein 1 (IRP1) in the cytoplasm is homologous to the mitochondrial aconitase, but with dual functions: the aconitase activity relying on 4Fe–4S integration and the cytoplasmic iron regulatory function. Under high-iron or low-oxygen conditions, the stability of Fe–S clusters maintains and the aconitase activity of IRP1 increases. Conversely, IRP1 without 4Fe–4S binds to the iron responsive element (IRE) of mRNA to regulate the translation of a series of iron-related genes.

Because of the sensitivity of Fe–S clusters to the redox environment, proteins containing Fe–S clusters are directly considered to be redox sensors (Crack and Le Brun 2018). In eukaryotes, IRPs are generally thought to be labile iron sensor and IRP2 is the main one *in vivo* (Meyron-Holtz *et al*. 2004). Early research found that IRP2 is degraded through an iron-dependent proteasome pathway, mediated by FBXL5 (F-box and leucine-rich repeat protein 5) to be ubiquitinated by ubiquitin ligase (SKP1-CUL1-ubiquitin-ligase) (Salahudeen *et al*. 2009; Vashisht *et al*. 2009). Recently, the oxygen-dependent regulation of IRP2 was revealed that the substrate binding region of the carboxyl-terminus of FBXL5 can bind 2Fe–2S. Under aerobic (normxia) conditions, oxygen obtains an electron from [2Fe–2S]^+^ to convert [2Fe–2S]^+^ into [2Fe–2S]^2+^, which strengthens the interaction of FBXL5 and IRP2 and mediates the degradation of IRP2 (Wang *et al*. 2020). The 2Fe–2S of FBXL5 was acquired from CIA machinery (Mayank *et al*. 2019). Therefore, FBXL5 is both an oxygen sensor and an iron sensor to regulate the cellular iron homeostasis in response to oxygen and iron availability (Rouault and Maio 2020; Ruiz and Bruick 2014).

## TRANSFER OF MITOCHONDRIAL Fe–S CLUSTERS

After synthesis, the Fe–S cluster must be transferred to Fe–S target protein promptly. Therefore, the carrier protein does not only bind to the Fe–S cluster scaffold protein ISCU and/or chaperone/co-chaperone proteins, but also binds to Fe–S recipients. Based on this idea, a series of studies on protein–protein interaction have been carried out for the discovery of the carriers, which do not need to tightly bind to scaffold or chaperone, nor the recipients in order to efficiently transfer Fe–S. It is just like hemoglobin as for oxygen with the effective association and disassociation. Therefore, better technical breakthroughs need to be developed to reach the goal.

Chaperone Hsc20 homolog Jac1 was found to bind to ISCU and to the co-chaperone HSC60 homolog Ssq1 in yeast (Andrew *et al*. 2006) as in *E. coli* (Silberg *et al*. 2004). Interaction of human HSC20 with ISCU and HSPA9 (HSC60 homolog) was confirmed (Uhrigshardt *et al*. 2010). A conformational change of the HSC20/HSPA9/ISCU complex occurs, which facilitates the transfer of Fe–S clusters from the scaffold protein ISCU to some recipients through HSC20 (Maio *et al*. 2014). Interestingly, the authors found that HSC20 can bind to a number of proteins with a LYR motif. The leucine (L) in the LYR motif can be replaced by isoleucine or alanine, tyrosine (Y) by phenylalanine or tryptophan, and arginine (R) by lysine. Proteins containing LYR motif potentially obtain 2Fe–2S or 4Fe–4S to either perform enzymatic functions, such as succinate dehydrogenase (4Fe–4S·SDHB), or act as a carrier protein GLRX5 (2Fe–2S·GLRX5) (Banci *et al*. 2014) for secondary transfer of Fe–S clusters to its downstream target proteins (Fig. 2).

It is commonly accepted that the primary transfer of Fe–S clusters depends on the assistance of chaperone system composed of HSC20/HSPA9 from ISCU to target proteins. The current work suggests that GLRX5, IND1, ISCA1, ISCA2, NFU1, BOLA3 and IBA57 are involved in Fe–S cluster delivery. According to the clinical manifestation of their mutations, it may be explained that GLRX5 is responsible for the transfer of 2Fe–2S and others are responsible for the transfer of 4Fe–4S. Of them, IND1 is important for the assembly of complex I (Calvo *et al*. 2010), probably, by being responsible for 4Fe–4S transfer to complex I. The clinical manifestations of ISCA1, ISCA2, NFU1, BOLA3 and IBA57 mutations are similar (https://www.omim.org/) and proteins show certain selective interaction (Beilschmidt *et al*. 2017), which suggest the hierarchical delivery of 4Fe–4S and selective targets. Very recently, *in vitro* experiments have demonstrated that ISCU and NFU1 directly interact, which facilitates the transfer of 4Fe–4S from ISCU to NFU1 (Cai *et al*. 2020). Though ISCA2–IBA57 heterotetramer complex containing two 2Fe–2S was proposed, how this complex would be involved in 4Fe–4S assembly or transfer *in vivo* remains unknown. Whether or not more carrier proteins are involved in the transfer and how these proteins participate in the proposed secondary, tertiary or later steps are interesting issues (Fig. 2).

## DISEASES RELATED TO MITOCHONDRIAL Fe–S CLUSTER SYNTHESIS AND TRANSFER

Fe–S protein widely exists in various organelles of cells and plays important physiological roles (Fig. 1). One of the well-known function of mitochondria is to provide chemical energy ATP to meet the needs of active biochemical reactions, but removal of mitochondrial DNA to collapse electron transport chain and to inhibit ATP production does not lead cell death, but sickness. On the contrary, cells cannot survive without mitochondrial Fe–S clusters biogenesis. Therefore, it seems that the synthesis of Fe–S clusters makes mitochondria essential (Lill *et al*. 2005) and Fe–S cluster as a cofactor is thought to be one of the oldest and relic characteristics of cells (Tsaousis 2019). Clinically, gene mutations, abolishing the synthesis or transfer of Fe–S clusters, often causes severe metabolic disorders (Table 1).

**Table 1:** Currently known diseases caused by mutations in genes related to mammalian mitochondrial iron–sulfur cluster biosynthesis and transfer

Protein	Functions	Associated diseases	Causes	Reference
Mitochondrial Fe–S biosynthesis associated genes				
Nfs1	Cysteine desulfurase, supplies inorganic sulfur to Fe–S clusters	Infantile complex II/III deficiency (IMC23D)	Missense mutation c.251G>A, p.Arg72Gln	Farhan *et al*. 2014; Land and Rouault 1998
ISD11	Binds cysteine desulfurase and helps free inorganic sulfur for Fe–S clusters.	Combined oxidative phosphorylation defects 19	Missense mutation c.203G>T, p.R68L	Adam *et al*. 2006; Lim *et al*. 2013
ACP	Interacts with ISD11 and stabilizes the function of NSF1 and the biosynthesis of Fe–S	Manic depressive illness (Bipolar disorder)	(SNP association)	Wellcome Trust Case Control 2007
Frataxin (FXN)	Involves Fe–S biosynthesis	Friedreich's ataxia (FRDA)	GAA trinucleotide repeat expansion in intron 1, suppresses the transcription of FXN and reduces its expression	Campuzano *et al*. 1996; Gellera *et al*. 2007
ISCU	Scaffold for Fe–S	Myopathy with lactic acidosis	Splicing mistake or splicing mistake and missense mutation c.149G> A, p.G50E	Kollberg *et al*. 2009; Mochel *et al*. 2008; Tong and Rouault 2006
FDX1	Electron transport intermediate for mitochondrial cytochromes P450 and Fe–S biosynthesis	–	–	Okamura *et al*. 1985; Shi *et al*. 2012
FDX2	Transfers electrons from NADPH to Fe–S biosynthesis via FDXR	Mitochondrial myopathy, episodic, with or without optic atrophy and reversible leukoencephalopathy (MEOAL)	Homozygote mutation c.1A>T, p.M1L or c.431C>T, p.P144L	Gurgel-Giannetti *et al*. 2018; Sheftel *et al*. 2010; Spiegel *et al*. 2014
FDXR	Receives electrons from NADPH, thus initiating the electron-transport chain serving mitochondrial cytochromes P450 and Fe–S biosynthesis	Auditory neuropathy and optic atrophy (ANOA)	Homozygote mutation c.916C>T, Arg306Cys or heterozygote mutation Gln419Ter/Leu215Val/Glu477Lys	Lin *et al*. 1990; Paul *et al*. 2017; Solish *et al*. 1988
Mitochondrial Fe–S transport associated genes				
HSPA9	Chaperone. It involves Fe–S transport	Congenital Sideroblastic anemia 4	Gene deletion	Kaul *et al*. 1995; Schmitz-Abe *et al*. 2015
		EVEN-PLUS syndrome	Homozygote mutation c.376C>T, p.R126W	Royer-Bertrand *et al*. 2015
HSC20	Cochaperone. It binds target proteins that contain the LYR motif	Congenital sideroblastic anemias (CSAs)	A paternally-inherited promoter variant (c.-134C>A) predicted to disrupt a conserved ETS transcription factor binding site, and a maternally-inherited frameshift (c.259dup, p.T87fs*27).	Maio *et al*. 2014; Sun *et al*. 2003; Uhrigshardt *et al*. 2010；Crispin *et al*. 2017
GLRX5	Fe–S carrier protein	Sideroblastic anemia 3	Mistaken splicing leads to low expression of GLXR5	Camaschella *et al*. 2007; Wingert *et al*. 2005
NFU1	Fe–S delivery to specific recipients	Multiple mitochondrial dysfunctions syndrome 1 (MMDS1)	c.545G>A,p.Arg182Gln; or homozygote mutation c.622G>T (p.Gly208Cys)	Al-Hassnan *et al*. 2015; Lorain *et al*. 2001; Navarro-Sastre *et al*. 2011
BOLA3	Fe–S delivery to specific recipients	Multiple mitochondrial dysfunctions syndrome 2 (MMDS2)	Single base-pair duplication c.123dupA, in exon 2, causing a frameshift that produces a premature stop codon; or homozygote missense mutation c.200T>A in exon 3, p.167N	Haack *et al*. 2013; Seyda *et al*. 2001; Zhou *et al*. 2008
IBA57	[Fe4-S4] assembly component for a subset of recipients	Multiple mitochondrial dysfunctions syndrome 3 (MMDS3)	Homozygote mutation c.941A>C,p.Gln314Pro	Ajit Bolar *et al*. 2013; Nilsson *et al*. 2009
		Autosomal recessive spastic paraplegia 74	Homozygote mutation c.678A-G	Lossos *et al*. 2015
ISCA2	Involves the maturation and assembly of Fe–S	Multiple mitochondrial dysfunctions syndrome 4 (MMDS4)	Homozygote mutation c.229G>A,p.G77S	Al-Hassnan *et al*. 2015; Sheftel *et al*. 2012
ISCA1	Involves the biosynthesis and assembly of Fe–S	Multiple mitochondrial dysfunctions syndrome 5 (MMDS5)	Homozygote mutation c.259G>A,p.E87K	Cozar-Castellano *et al*. 2004; Shukla *et al*. 2017
IND1	Mitochondrial translation; complex I assembly	Childhood-onset mitochondrial encephalopathy and complex I deficiency	c.166G>, p.G56R with deletion spanning exons 1-4. A second substitution in intron 9 (c.815-27T> C) resulting in aberrant splicing	Calvo *et al*. 2010; Sheftel *et al*. 2009
ABCB7	Component of the mitochondrial export machinery	X-linked sideroblastic anemia with cerebellar ataxia (XLSA/A)	Several mutations close to or in transmembrane domains of the ABC transporter	Allikmets *et al*. 1999; Bekri *et al*. 2000; Savary *et al*. 1997

### Defects related to Fe–S cluster synthesis

It is known to date that the mutations of the core components, NFS1, ISD11, ISCU and FXN, cause human diseases (Table 1). These include infant mitochondrial complex II and III deficiency caused by *NFS1* mutation (Farhan *et al*. 2014), combined oxidative phosphorylation deficiency by *ISD11* mutation (Lim *et al*. 2013), Friedrich's ataxia by *FXN* mutation (Campuzano *et al*. 1996), and *ISCU* mutation (Mochel *et al*. 2008). Mammalian ferredoxin and ferredoxin reductase (FDX / FDXR) provide electrons for Fe–S synthesis (Shi *et al*. 2012). *FDX2* mutation has been found to produce sporadic mitochondrial myopathy with or without optic atrophy and reversible leukoencephalopathy (MEOAL) (Gurgel-Giannetti *et al*. 2018; Spiegel *et al*. 2014). *FDXR* mutation raises auditory neuropathy and optic atrophy (ANOA) (Paul *et al*. 2017).

#### Infantile mitochondrial complex II/III deficiency $( $IMC23D$) $

Human *NFS1* was originally identified through homology comparison. *NFS1* is located on chromosome 20q11.22 and encodes cysteine desulfurase NFS1 required for Fe–S cluster synthesis (Land and Rouault 1998). Its mutation was first reported in 2014 (Farhan *et al*. 2014). Exon sequencing revealed that the mutation of G→A resulted in the substitution of the amino acid Arg72Gln, caused IMC23D in a mode of autosomal recessive inheritance with clinical symptoms of multiple organ failure during infancy (Farhan *et al*. 2014). The very early onset of the disease indicates that NFS1 is an essential gene and has an irreplaceable role in Fe–S biosynthesis.

#### Combined oxidative phosphorylation defects 19 $( $COXPD19${\text{)}} $

Human *ISD11* was identified in 2006 initially by a systematic approach to characterize essential proteins involved in Fe–S biogenesis in yeast and then by homologue searching in human genome (Adam *et al*. 2006). The position of *ISD11* on the chromosome is 6p25.1. The encoded protein has a highly conserved LYR motif at the N-terminus and participates in mitochondrial Fe–S synthesis by interacting with NFS1 and ACP. The homozygous missense mutation 203G>T of *ISD*11 was found in two patients from two families both with consanguineous marriages in one big family, resulting in the substitution of the amino acid Arg68Leu, which is the genetic basis of COXPD19 (Lim *et al*. 2013). The disease is autosomal recessive with deficiency of the activities of mitochondrial complexes I, II and III in the liver and muscle. A number of enzymatic activities decrease and the expression of Fe–S proteins also decreases significantly (Lim *et al*. 2013).

#### Friedreich's ataxia $( $FRDA$) $

The genetic basis of Friedreich's ataxia was finally determined after a long-term investigation from Mendel analysis and gene mapping to DNA sequencing (Campuzano *et al*. 1996). The causative gene *FXN*, located on chromosome 9q21.11, encodes a 210 amino acid precursor, which is further processed through a two-step cleavage to be a mitochondrial mature form (Schmucker *et al*. 2008; Xia *et al*. 2012). With the development of epigenetics, scientists have discovered that the repeated expansion of the GAA repeats (200–1700+) in the first intron of *FXN* results in the formation of heterochromatin (Saveliev *et al*. 2003), thereby inhibits *FXN* transcription. The number of GAA repeats is closely correlated to the level of *FXN* expression and the age of onset of the disease, *i.e*. more GAA expansion, less expression, and earlier onset of the disease (Koeppen 2011). The reduced expression is the main reason counting for the occurrence of the disease. Other types of mutations have also been discovered, such as multiple missense mutations and nonsense mutations, to make FXN dysfunction (Gellera *et al*. 2007). This autosomal recessive disease is characterized most often by the progressive ataxia of limbs and gait, dysarthria, cardiomyopathy and an increased rate of diabetes mellitus (Koeppen 2011). Most patients die of heart failure in young adult life (Tsou *et al*. 2011).

#### Myopathy with lactic acidosis

In 2000, human *ISCU*, located on chromosome 12q23.3, was cloned and functional assays were performed (Tong and Rouault 2000). This protein has an extra-mitochondrial isoform as human *NSF1* with the same function as within mitochondria to maintain the steady state of the Fe–S clusters outside mitochondria (Li *et al*. 2006; Tong and Rouault 2006). The mutation 7044G>C of *ISCU* was first reported, which resulted in a new splicing site, mostly, in skeletal muscle to produce a truncated and non-functional isoform, therein, to reduce the levels of canonical *ISCU* mRNA and functional protein (Mochel *et al*. 2008). Why the cleavage site formed by this mutation is not used at high frequency in other tissues is an interesting question. Other type of mutation 149G>A in exon 3 of *ISCU* in two patients was reported, resulting in the amino acid substitution G50E (Kollberg *et al*. 2009). The patient is a heterozygote with above two mutations, one allele being spliced erroneously, another expressing a not-working protein. The consequence is inadequate mitochondrial Fe–S clusters synthesized. The typical clinical features are muscle weakness, dysplasia and wasting (Mochel *et al*. 2008). In the early phase, wheelchair assistance is required, and in the later phase, severe spine flexion and hunchback occur (Kollberg *et al*. 2009).

### Fe–S transfer defect-associated diseases

To date, the transfer of Fe–S clusters is not understood well as the synthesis. Genes involved in Fe–S cluster transfer seem to be more than ones in Fe–S biogenesis. How the biogenesis machinery guides selection of the specific Fe–S recipients is a challenging question. The clinical disorders always provide valuable clues.

#### Congenital sideroblastic anemia 4 $( $CSA4$) $ and EVEN-PLUS syndrome

HSPA9/HSC20 are currently regarded as key chaperones in the transfer of Fe–S clusters, responsible for the primary transfer of the nascent Fe–S cluster (Fig. 2) (Maio *et al*. 2020). Human *HSPA9* is located on chromosome 5q31.1 and encodes a protein localized in mitochondria, cytoplasm and endosome (Kaul *et al*. 1995). The different clinical phenotypes induced by various mutations of the same gene might due to the multiple localization, which makes HSPA9 serve different Fe–S recipients. For instance, congenital sideroblastic anemia 4 (CSA4) was first discovered due to a frameshift mutation caused by the 2 bp deletion in *HSPA9* gene, resulting in a premature stop codon (Schmitz-Abe *et al*. 2015). The hyperplasia of erythroblasts in bone marrow with the appearance of ringed sideroblasts and ineffective erythropoiesis are found to be the pathological features for CSA4. In the same year, EVEN-PLUS syndrome (EVPLS) was revealed to be caused by *HSPA9* gene mutations (c.376C>T and 383A>G or 882-883delAG), which lead to amino acid substitution Arg126Trp, Tyr128Cys, or early termination, respectively (Royer-Bertrand *et al*. 2015). This mutation-induced disorder is similar to the disease caused by mutation of another mitochondrial chaperone *LONP1* in clinical manifestations of vertebral and epiphyseal aplasia, microtia, flat nose and skeleton deformity. HSC20 mutation might induce congenital sideroblastic anemia (Crispin *et al*. 2017), which result has not been published.

#### Congenital sideroblastic anemia 3 $( $CSA3$) $

Besides (co)chaperones, GLRX5, IND1, BOLA3, NFU1, IBA57 and ISCA1/2 are also considered to be involved in Fe–S clusters transfer. The first recognized disease associated with Fe–S cluster transfer is CSA3, caused by *GLRX5* mutation (Camaschella *et al*. 2007). By sequence alignment with yeast homologous, human *GLRX5* gene was identified. *GLRX5* is located on chromosome 14q32.13. The function of GLRX5 was reported to link Fe–S clusters and heme synthesis (Wingert *et al*. 2005). *GLRX5* mutation (homozygous c.294A>G) disturbs intron 1 splicing and drastically reduces *GLRX5* RNA and protein levels (Camaschella *et al*. 2007). Recently, more *GLRX5* mutations were found (missense mutation K101Q and L148S) in patients with reduced FECH activity (Liu *et al*. 2014), suggesting that FECH might be the target protein of GLRX5 to acquire 2Fe–2S (Fig. 2).

#### Multiple mitochondrial dysfunctions syndrome $( $MMDS$) $

MMDS currently has six subtypes (MMDS1–6), which are caused by the mutation of six different genes, which are *NFU1*, *BOLA3*, *IBA57*, *ISCA2*, *ISCA1* and *PMPCB*. As indicated by the names of the diseases, multiple organs are affected at early age with onset from infancy, showing the vital functions of these genes. Of them, the first five genes are, not only functionally, but also physically, related (Cameron *et al*. 2011; Sheftel *et al*. 2012). However, MMDS6 reported recently is regarded as a result of reduced mitochondrial processing protease activity, accompanied with decreased FXN mature form (Vogtle *et al*. 2018), which is very important in early steps of Fe–S biosynthesis.

(A) Multiple mitochondrial dysfunctions syndrome 1 (MMDS1)

The human *NFU1* gene was originally cloned from a cDNA library and mapped to chromosome 2p13.3 and the protein has a mitochondrial localization signal peptide, a conserved N-terminal domain, and a variable C-terminus (Lorain *et al*. 2001). The mutation c.545G>A results in amino acid substitution Arg182Gln (Cameron *et al*. 2011), and c.622G>T results in substitution Gly208Cys (Navarro-Sastre *et al*. 2011), leading to the autosomal recessive MMDS1. Most patients die before two years old. Based on the interaction between ISCU and NFU1 (Cai *et al*. 2020), it is speculated that the formed 2Fe–2S on scaffold ISCU fuse into 4Fe–4S on NFU1 within mitochondria (Tong *et al*. 2003) to be delivered to a apoprotein or to a 4Fe–4S carrier protein for further transfer (Fig. 2). Therefore, NFU1 may function as a scaffold protein of 4Fe–4S in the early step of 4Fe–4S transfer. It may explain that mutation in this gene cause severe defects in the development of infants.

(B) Multiple mitochondrial dysfunctions syndrome 2 (MMDS2)

Human *BOLA3* gene was named from the homologue of *E.coli* (Zhou *et al*. 2008). *BOLA3* is located on chromosome 2p13.1. The autosomal recessive disease caused by *BOLA3* mutation was reported (Seyda *et al*. 2001). A repeat A (123dupA) in the exon of the *BOLA3* gene was identified causing a frame shift that produces a premature stop codon (p.Glu42Argfs(*)13). The patients were characterized by reduced pyruvate dehydrogenase and respiratory chain complex (Cameron *et al*. 2011). The clinical manifestations of this disease are very similar to MMDS1 for both of the mutation in *BOLA3* and *NFU1* were simultaneously detected in one screening for one disease (Cameron *et al*. 2011), suggesting that both closely function in the early steps of Fe–S cluster transfer.

(C) Multiple mitochondrial dysfunctions syndrome 3/4/5 (MMDS3/4/5)

MMDS3/4/5 are autosomal recessive inherent diseases. The patient with *IBA57* mutation-induced MMDS3 was born with severe hypotonia and lactic acidosis, and mitochondrial defects, particularly the deficient activities of complex I, II and IV (Ajit Bolar *et al*. 2013). The mutation c.941A>C in the patient resulted in amino acid substitution Gln314Pro, which made IBA57 protein unstable and easy to be hydrolyzed. Later, more mutants were found (Lossos *et al*. 2015).

MMDS4 was first characterized in a consanguineous marriage (Al-Hassnan *et al*. 2015). The mutation of *ISCA2* gene c.229G>A results in amino acid substitution Gly77Ser, which is within the Fe–S binding domain, leading to mitochondrial depletion and reduced complex I activity. The patients show the loss of roll-over ability even at age of four with spastic lower limbs and optic atrophy, but normal complete blood count.

The first mutation of *ISCA1* in patients with MMDS5 was found by exon sequencing and the same homozygous mutation in two individual families, both of which were in consanguineous marriages within a big family, suggesting the possibility of a founder effect (Shukla *et al*. 2017). Gene mutation c.259G>A leads to amino acid substitution Glu87Lys, which leads to the instability and reduced protein level of ISCA1. The inherent mode and clinical manifestations are very similar to other four MMDS.

The human *IBA57*, *ISCA2* and *ISCA1*, located on the chromosomes 1q42.13, 9q21.33, and 14q24.3, respectively, encode mitochondrial proteins, which are seemly involved in 4Fe–4S assembly/transfer and are essential to electron transport chain and mitochondrial function (Cozar-Castellano *et al*. 2004; Nilsson *et al*. 2009; Sheftel *et al*. 2012).

#### Childhood-onset mitochondrial encephalopathy and complex I deficiency

Human *IND1* gene is located on chromosome 14q12, and the encoded protein contains mitochondrial targeting sequence at the N-terminus. IND1, including a highly conserved nucleotide binding domain and Fe–S binding domain, is a component of complex I and in charge of the assembly and acquirement of Fe–S clusters in complex I (Sheftel *et al*. 2009). The mutation c.166G>A of IND1, resulting in the substitution of Gly56Arg, was first found to lead to mitochondrial complex I deficiency, nuclear type 21. The main clinical symptom in these patients is developmental delay, especially defective motor ability, accompanied by myopathy, ataxia, and language impairment (Calvo *et al*. 2010). All patients developed motor problems due to ataxia in the first years of life (Kevelam *et al*. 2013).

## PERSPECTIVES AND BASIC QUESTIONS IN THE FIELD OF Fe–S CLUSTER RESEARCH

As prosthetic groups, Fe–S clusters participate in various bio-activities and are indispensable components, particularly, in eukaryotes. Even in highly differentiated mature erythrocytes, there are no newly synthesized mitochondrial Fe–S clusters, it is still thought that cells must prepare enough Fe–S clusters, at least, 2Fe–2S for FECH to insert iron into protoporphyrin IX before mitochondrial extrusion. Yet, we do not know if extra-mitochondrial Fe–S machinery would take some roles, not depending on mitochondrial machinery, for other cellular Fe–S proteins in the late stage of erythrocytes.

Although the core components of the Fe–S biosynthesis machinery are assembled with the ratio of NFS1:ISD11:ACP:ISCU:FXN = 2:2:2:2:2, any mutation of these genes causes disease in a recessive mode of inheritance no matter whether one mutation causes the functional substitution of amino acids or the mutation reduces the protein expression. This phenomenon suggests that, (1) The formation of the two 2Fe–2S on two scaffold ISCU in one complex is two independent events; (2) The formation of 4Fe–4S is not just fusion of two 2Fe–2S from the same complex of the machinery, but maybe from two individual complexes; (3) The formation of 4Fe–4S attributes to other proteins, including NFU1. Therefore, some questions are raised: Does ISCU act as a scaffold for both 2Fe–2S and 4Fe–4S? Does 4Fe–4S need its own scaffold for the formation of 4Fe–4S? Are there one or more scaffold proteins? The function of FXN is not yet very clear in our eyes since the patients of FRDA have no systemic anemia, indicating that 2Fe–2S containing FECH is mildly, if some, affected. Although FXN is believed to play an important role as an allosteric activator of NFS1 during the synthesis of Fe–S clusters, we envision that FXN also plays a role in the formation of 4Fe–4S. The function of ACP in the synthesis step is unclear, and no very-sure clinical patients have been found so far (Wellcome Trust Case Control 2007). In the synthesis process of Fe–S, what protein supplies iron ions is an unsolved question, the answer to which scientists are looking for. Initially, ISCA1 and 2 were proposed to have the function of donating iron (Ding *et al*. 2004), but later ISCA1/2 in mammalian cells are thought, more likely, to be a protein that delivers 4Fe–4S (Sheftel *et al*. 2012).

Two typical diseases, caused by mutations of Fe–S delivery genes, are encephalopathy/myopathy and hematopoietic disorder, suggesting that delivery of 2Fe–2S and 4Fe–4S is selectively guided by different carriers as shown in Fig. 2. Nowadays, GLRX5 is considered to be a carrier of 2Fe–2S for a set of targets, while NFU1, BOLA3, IBA57, and/or ISCA1/2 to be responsible for the delivery of 4Fe–4S. How they deliver Fe–S and what proteins are their individual targets remain elusive. The mutation of HSPA90 is complicated, causing two distinct disorders, sideroblastic anemia and EVEN-PLUS syndrome, with dominant or recessive modes of inheritance. The outcomes from the mutation suggest that HSPA90 functions in multiple steps of Fe–S cluster transfer.

In summary, there are still many unknown issues in the synthesis and transfer of Fe–S clusters in mammalian cells. Among them, the function of FXN is specially of interest. Iron donor of Fe–S cluster biogenesis is missing. The very speculated hypothesis is proposed whether the transfer of 2Fe–2S and 4Fe–4S is hierarchically processed (Fig. 2), which is presented for discussion in this field.

## Conflict of interest

Wenxin Zhang, Li Xu, Hongting Zhao and Kuanyu Li declare that they have no conflict of interest.
